# Results at seven years after the use of intracamerular cefazolin as an endophthalmitis prophylaxis in cataract surgery

**DOI:** 10.1186/1471-2415-12-2

**Published:** 2012-01-24

**Authors:** Pedro Romero-Aroca, Isabel Méndez-Marin, Merce Salvat-Serra, Juan Fernández-Ballart, Matias Almena-Garcia, Javier Reyes-Torres

**Affiliations:** 1 Professor of Ophthalmology. Ophthalmology Service. Hospital Universitari Sant Joan, IISPV, Universidad Rovira i Virgili, Reus (Spain; 2 Ophthalmology Service. Hospital Universitari Sant Joan, IISPV, Universidad Rovira i Virgili, Reus (Spain; 3 Department of Basic Sciences, University Rovira i Virgili, Tarragona (Spain). CIBER Fisiopatología de la Obesidad y Nutrición (CIBEROBN), Instituto de Salud Carlos III (ISCIII), (Spain

**Keywords:** endophthalmitis, cataract surgery, cefazoline, prophylactic ophthalmic surgery, cephalosporines

## Abstract

**Background:**

To evaluate results after seven years using prophylactic intracameral cefazolin for the prevention of endophthalmitis in cataract surgery.

**Methods:**

A prospective, observational study of all patients submitted to cataract surgery over the period January 1996 to December 2009. All cases of postoperative endophthalmitis over that period were reviewed. The patients were classified in two groups: Group 1 (11,696 patients) operated on between January 1996 and December 2002, Group 2 (13,305 patients) between January 2003 and December 2009 (in whom a 1 mg/0.1 bolus of intracameral cefazolin was instilled).

**Results:**

During the study period, 76 cases of endophthalmitis were observed in Group 1, and seven in Group 2. The rate of postoperative endophthalmitis reduced from 0.63% to 0.05% with a cefazolin injection. The relative risk (RR) for endophthalmitis in Group 1 against group 2 was 11.45 *[95% CI 5.72-22.84, p < 0.001]*.

**Conclusions:**

An intracameral bolus injection of cefazolin (1 mg in 0.1 ml solution) at the conclusion of the cataract surgery significantly reduced the rate of postoperative endophthalmitis.

## Background

Endophthalmitis remains a serious complication after cataract surgery, although prophylactic measures introduced in recent years have reduced the number of patients with this complication.

Currently there are two streams of opinion towards endophthalmitis prophylaxis, the use of fourth-generation quinolones (gatifloxacin and moxifloxacin) topically [1-4], or the introduction of intracameral cephalosporins, the latter being cefuroxime (a second generation cephalosporin), which is the most widely used and accepted [5-10]. However, our study group [11], as well as Garat et al [12,13], prefers the use of cefazolin (a first generation cephalosporin). Having previously studied the bacteria that cause endophthalmitis in our environment most frequently, we prefer cefazolin because of its higher frequency of gram-positive bacteria in our medium, and because it best covers infections by such bacteria. Furthermore, cefazolin shows no corneal toxicity at doses of 1 mg or 2 mg; its toxicity was established when doses of 5 mg or more were injected. We consider the risk of an infection caused by a cefazolin-resistant bacterium low, based on the bacteria cultured since 1994 and their antibiogram. We consider there not to be any risk of coverage of gram-negative bacteria by cefazolin, and its incidence in endophthalmitis was low in our Health Care District.

Our two groups are included in the Barcelona Endophthalmitis Group (GEB), formed by 38 public and private Hospitals in Catalonia (Spain), who have been studying the epidemiological factors of postoperative endophtalmitis and assessing the prophylaxis and treatment of endophtalmitis in our country since 2000. Appendix 1 gives a list of GEB participating hospitals and ophthalmologists.

This study will present the results obtained after seven years of using intracameral cefazolin after cataract surgery at doses of 1 mg in 0.1 ml.

## Methods

Since 1996, there has been an ongoing registration of all postoperative endophthalmitis patients at Hospital St Joan (Table 1). All cases of postoperative endophthalmitis were studied in the epidemiological unit of the hospital in order to determine their origin.

**Table 1:** Description of cases of endophthalmitis since 1996

Group 1 1996-2002								
	**1996**	**1997**	**1998**	**1999**	**2000**	**2001**	**2002**	**Total**

Cases*	8	9	9	11	12	13	14	76

Percentage	0.637%	0.613%	0.629%	0.664%	0.634%	0.687%	0.692%	0.649%

Number of operations**	1256	1467	1430	1656	1892	1972	2023	11696

**Group 2 2003-2009**								

Cases*	1	1	1	0	2	1	1	7

Percentage	0.058%	0.052%	0.055%	0.000%	0.101%	0.050%	0.049%	0.052%

Number of operations**	1707	1911	1812	1874	1967	1997	2037	13305

Cases* = number of cases of endophthalmitis (proven + unproven) occurring each year

Patients** = Number of cataract interventions carried out each year

### Design

A prospective observational study. The population comprised all patients submitted to un-combined cataract surgery in the period from January 1996 to December 2009. All cases of postoperative endophthalmitis were related. Only patients with phacoemulsification were included in the study in order to reduce any possible bias introduced by the technical cataract surgery. Technical surgery includes a sutureless, clear corneal incision of 3.2 mm using a venturi (Millenium, Bausch Lomb^®^) phaco unit.

For all patients included in our hospital, the prophylactic measures for reducing the bacterial flora of the conjunctiva since 1996 were:

• Topical 10% povidone-iodine in the skin of peri-orbital region

• Topical 5% povidone-iodine on the conjunctiva and eyelashes for a minimum of 1 minute

• Draping of the peri-orbital region and eyelashes

• Postoperative use of eye drops with tobramycin 3.00 mg/mL associated to dexametasone 1.00 mg/mL instilled every 4 hours (these eye drops were gradually tapered after 1 week), and diclofenac every 6 hours. These drops were continued at week 3, and the topical diclofenac was used for 1 month after surgery.

The patients were classified in two groups:

• Group 1 included patients operated on from January 1996 to December 2002 (11696 patients), during which time intracameral cefazolin was not used.

• Group 2 included patients operated on from January 2003 to December 2009 (13305 patients) when we used 1.00 mg/0.1 mL intracamerular cefazolin was used at the end of cataract surgery.

### Antibiotic selection

The choice of cefazolin as an intracameral antibiotic was based on bacterial and antibiogram studies in endophthalmitis cases since 1996 (Table 2), and the antibiogram (Table 3).

**Table 2:** Bacteria cultured in cases of endophthalmitis in the two groups.

	Number of cases (percentage) 1996-2002	Number of cases (percentage) 2003-2009
Negative cultures	26 (34.21%)	2 (28.57%)

*Gram positive (96.00%)*		

*Staphylococcus epidermidis*	37 (74.00%)	

*Staphylococcus aureus *methicillin sensible	5 (10.00%)	

*Streptococcus pneumoniae*	2 (4.00%)	

*Streptococcus* *viridans*	1 (2.00%)	

*Bacillus spp*	3 (6.00%)	

*Corynebacterium*		1 (14.28%)

*Gram negative (4.00%)*		

*E Colli*	1 (2.00%)	

*Serratia marcensens*	1 (2.00%)	

*Klebsiella pneumoniae*		1 (14.28%)

*Pseudomona aeroginosa*		1 (14.28%)

*Proteus mirabilis*		2 (28.57%)

**Table 3:** Sensitivity to antibiotics of the most frequent bacteria in our Area in 2002

Germen	Cefazolin	Cefuroxime	Levofloxacin	Vancomicyn
Gram positive				

*Staphilococcus Aureus*	100% (≤2 ≥4)*	96% (≤2 ≥4)	65% (≤0.5 ≥8)	66% (≤4 ≥32)

*Staphilococcus Aureus* MARSA	54% (≤2 ≥4)	48% (≤2 ≥4)	17.64% (≤0.5 ≥8)	99,02% (≤4 ≥32)

*Staphilococcus Epidermidis*	100% (≤2 ≥4)	97% (≤2 ≥4)	27% (≤0.5 ≥8)	97% (≤4 ≥32)

*Streptococcus Pneumoniae*	96% (≤2 ≥8)	79.15% (≤0.12 ≥2)	89% (≤2 ≥8)	100% (≤1 -^f ^* )

Gram negative				

*E. Colli*	85% (≤4 ≥32)	87% (≤4 ≥32)	71% (≤0.5 ≥8)	90% (≤4 ≥32)

*Pseudomona Aeroginosa*	18% (≤4 ≥32)	15% (≤4 ≥32)	65% (≤2 ≥8)	65% (≤4 ≥32)

*Proteus* *Mirabilis*	79% (≤4 ≥32)	82% (≤4 ≥32)	70% (≤0.5 ≥8)	100% (≤4 ≥32)

*Klebsiella Pneumoniae*	75% (≤4 ≥32)	79% (≤4 ≥32)	100% (≤0.5 ≥8)	95% (≤4 ≥32)

(≤2 ≥4)* = MIC (minimum inhibitory concentration) = Critical concentrations in μg/ml (critical concentration of susceptibility, critical concentration of resistance), according to the recommendations of MENSURA to the interpretation of antibiogram.

-^f ^* = Unknown, we do not know the mechanisms of resistance, or the measure for resistance.

Table 3 was created from the analysis of an antibiogram of 14,626 cultures obtained from samples of patients with community- and hospital- acquired infections (urinary infections, pneumonia, sepsis, etc.), which occurred during 2002 in the population serviced by our Health Care District. In the table we presented only the results obtained with the bacteria cultured in endophthalmitis observed in our area, and compared with the prophylactic antibiotics most frequently used for endophthalmitis in Spain (cefazolin, cefuroxime, vancomicyn, and levofloxacin). At this point we should point out that in our Hospital in cases of allergy to cephalosporines we used vancomicyn.

The dose of cefazolin (1 mg/0.1 ml) was based on our calculations that this anterior chamber concentration of cefazolin exceeded the minimum inhibitory concentration (MIC) for susceptible bacteria.

### Inclusion criteria

Patients in our dependent Health care district who were submitted to cataract surgery by phacoemulsification by clear cornea incision.

### Exclusion criteria

Patients admitted for extracapsular cataract surgery. Patients allergic to cephalosporins and to whom we gave intracamerular vancomicyn.

### Ethics

Local ethics committee approval the study CEIC HUSJ-2002-DEC-1022

### Definition of acute postoperative endophthalmitis

Following the criteria [14] in the Endophthalmitis Vitrectomy Study (EVS), the ophthalmologist diagnosed presumed acute endophthalmitis. If a positive culture of a vitreous sample was obtained, we defined the case as a proven acute endophthalmitis. In all proven and unproven cases, the patients had swollen lids, pain and an opaque vitreous.

### Microbiological method

Vitreous samples obtained by the ophthalmologist were immediately processed, then a Gram stain was carried out and the sample cultivated in petri dishes. Antibiogram susceptibility was measured according to the criteria laid down by the Spanish Committee for the Standardization of the Sensitivity and Resistance to Antimicrobials (MENSURA) [15-17] which includes members of the Spanish Society of Chemotherapy and the Spanish Society for Infectious Diseases and Microbiology [15]. This is a body with responsibilities similar to those of the National Committee for Clinical Laboratory Standards [18].

Statistical analyses for descriptive statistics were performed using SPSS statistical software (version 17.0). The data obtained was analysed with frequency and descriptive statistics. Values are expressed as mean ± SEM, and statistical significance was determined using the Student's t-test for paired data.

## Results

### Demographic results in the two groups of patients

Group 1. Formed by patients without intracameral instillation of cefazolin. This group included a total of 11,696 patients, with a median age of 69.8 ± 7.55 years (53-89 years); a total of 6785 (58.01%) were females. Group 2. Formed by patients with intracameral instillation of cefazolin at doses of 1 mg/0.1 mL. This group included a total of 13,305 patients, with a median age of 66.17 ± 7.83 years (53-81 years); a total of 7717 (58.00%) were females. The difference between groups was not statistically significant in the Student's t-test.

### Endophthalmitis cases

In Group 1 there were 76 postoperative endophthalmitis cases, at a median elapsed time of 5.37 ± 2.33 days after surgery. In 16 (69.77%) cases, cultures were positive for the following sub-classified gram-positive cultured bacteria: 9 cases (39.13%) of *Staphylococcus epidermidis*, 4 cases (17.39%) of *staphylococcus aureus *and 2 cultures (8.70%) positive for *Streptococcus spp*. The gram-negative bacteria gave a culture positive for *Klebsiella pneumoniae *(4.35%). Group 2, there were seven postoperative endophthalmitis cases, oat a median elapsed time of 5.41 ± 2.29 days after surgery. The statistical study of differences in respect to first group were significant at p < 0.001, RR: 11.45 [95% CI 5.72-22.84].

The bacteria cultured were:

• The first case involved patients with diabetes mellitus type II, treated with insulin for 30 years, with macro vascular disease with symptoms of intermittent claudication. The culture was positive for a Gram-negative bacterium (*Klebsiella pneumoniae*).

• The second case was a patient who lived alone and who had serious social problems; the patient also had domestic animals at home. The culture was positive for a Gram-positive anaerobic bacterium (*Corynebacterium*).

• The third case, positive for *Proteus mirabilis*, occurred in a 68-year-old woman with type II diabetes mellitus of long evolution (22 years), with poor glycaemic control and peripheral vascular macroangiopathy.

• The fourth case, positive for *Proteus mirabilis*, occurred in a 77-year-old man with type II diabetes mellitus and poor glycaemic control.

• The fifth case, positive for *Pseudomona aeroginosa*, occurred in a 73-year-old man with viral C-hepatitis.

• Negative cultures: two cases of negative cultures (28.57%) were observed in this period of time.

In the groups of patients that received intracamerular cefazolin, there were no cases observed of toxic effect at the corneal or retina levels, nor was there any hypersensitivity reaction.

### Statistical analysis

The relative risk for presenting with endophthalmitis in Group 1 compared with Group 2 was 11.45 [95% CI 5.72-22.84, p < 0.001]. When limiting the analysis to proven cases (50 cases in Group 1, against 5 cases in Group 2), the estimators of the relative risk were 14.07 [95% CI 7.68 - 24.48, p < 0.001].

### Visual acuity

1. In Group 1 (period 1996-2002) 38 over 76 patients (50%) had a final visual acuity (VA) over 0.1 and six patients (7.9%) had VA > 0.4 on the Snellen charts. There were ten patients with no light perception (four cases with negative culture, one case produced by *Seratia marcensens*, and five cases of *Streptococcus pneumoniae*). *Staphilococcus epidermidis *in 18 cases caused a final VA between 0.1 and 0.4, and in 14 cases a final VA between light perception and 0.1; the *Staphilococcus aureus *predominantly produced a final VA between light perception and 0.1 (four cases) and only one case with final VA > 0.4 on the Snellen charts (Table 4).

**Table 4:** Final visual acuity in patients with endophthalmitis.

Group 1 (1996-2002)				
Culture	No light perception	Light perception to < 0.1	0.1 to 0.4	> 0.4

Gram positive	5 (*Strp. Pneumoniae, Bacillus spp*.)	18*	19**	6***

Gram negative	1 (*Serratia marcensens*)		1(*E. Colli*)	

Negative culture	4	10	12	

**Group 2** **(2003-2009)**				

Culture	No light perception	Light perception to < 0.1	0.1 to 0.4	> 0.4

Gram positive	1 (*Corynebacterium*)			

Gram negative	2(*Klebsiella pneumoniae, Pseudomona aeroginosa*)	2 (*Proteus mirabilis*)		

Negative culture	1	1		

18* = 14 cases of *Staph. Epidermidis *and 4 cases of *Staph. Aureus*

24** = 18 cases of *Staph. Epidermidis *and 1 case of *Staph. Aureus*

6*** = One case of *Strp. Viridans*, and four cases of *Staph. Epidermidis*

NA = No patients with negative culture endophthalmitis in Group 1, and no patients in Group 2 with Gram-positive bacteria endophthalmitis.

2. In Group 2 (period 2003-2009) four patients had final VA of no light perception (two cases with gram-negative bacteria, one case with negative culture and one case produced by gram-positive corynebacterium). In the other three cases, the final VA was inferior to 0.1 on the Snellen charts (two cases with gram-negative bacteria and one with a negative culture) (Table 4).

## Discussion

In our health care district we had a high level of endophthalmitis. Previously, the intracamerular use of antibiotics, as we point out in the present study in group 1, lead to the incidence of endophthalmitis at a rate of 0.649%. This value is higher than that reported in other countries [2,6,8,14], but the incidence in Spain was also higher than other study groups, Garcia-Saenz et al [19] found an incidence of 0.59% (95% CI, 0.50%-0.70%) between January 1999 and September 2005, and Garat et al showed an incidence of 0.422% (95% CI 0.279-0.613) in their studies [12,13]. Because our centre had an excessive number of endophthalmitis cases despite using all means of regular prophylaxis (a sterile ophthalmology operating room, povidone-iodine in skin (at 10%) and conjunctival sac (at 5%) with few surgical intraoperative complications etc.), we decided to use intracamerular antibiotics after cataract surgery.

Peyman et al. published the first report of successful prophylactic bolus injections of antibiotics into the anterior chamber in 1977 [20]. Despite the efficacy of the injections, the technique later becomes forgotten about. It is well-established that the source of most infecting agents is the patients' ocular flora; the most frequently reported being bacteria gram-positive, coagulase-negative, or positive staphylococcus.

Swedish physicians have pioneered the use of intracameral cefuroxime since 2002, with excellent outcomes in 400,000 surgical interventions. Montan et al. published the efficacy of cefuroxime 1 mg intracameral [5,6], a practice that has lowered the rate of postoperative endophthalmitis from 0.26% to 0.06%. The large, prospective, multi-centred study, the European Society of Cataract Refractive Surgeons (ESCRS) confirmed the Swedish experience, finding that an injection of cefuroxime at the end of the surgery reduced endophthalmitis rates to just 0.05% [8-10].

We chose to use cefazolin, which is recommended by the Department of Microbiology and the Infectious Diseases Committee of our Hospital, rather than cefuroxime because it is a first generation cephalosporin and has a wide range of activity against gram-positive bacteria in our Health Care District, rather than a second-generation cephalosporin, such as cefuroxime. A study in our Health Care District by Vila-Corcoles et al [21], showed an increased resistance of *Streptococcus pneumoniae *to cefuroxime but not to cefazolin.

It is interesting to observe in Table 3 that cefazolin and cefuroxime present a similar antibiotic resistance pattern, with regard to both gram-positive and gram-negative bacteria. Therefore, we still believe that cefazolin is a good option as a prophylaxis for endophthalmitis, and its substitution for cefuroxime is not important for reducing the number of cases of endophthalmitis. However, by using cefazolin, there is still a risk of infection by gram-negative bacteria, which is why the majority of proven cases of endophthalmitis in Group 2 (4/5 cases were gram-negative) were caused by this type of bacteria. This is clearly a limitation in the use of cefazolin as a prophylaxis for endophthalmitis.

The lack of availability of eye-drops of fourth-generation quinolones in Spain is the most obvious reason for the preference for intracamerular antibiotics in the prophylaxis of endophthalmitis. Since June 2010, a moxifloxacin eye-drop has been available in Spain, with more possibilities of prophylaxis.

Results show that final VA was worse in Group 2 than in group 1. The explanation for this can be found in the type of bacteria that cause endophthalmitis. We found *Corynebacterium*, *Klebsiella pneumonia*, *Pseudomona aeroginosa*, and *Proteus mirabilis *in group 2. All these bacteria were poorly sensitive to cefazolin. Negative culture was observed in a 34.21% of cases in group 1 and 28.57% in Group 2, similar to other studies, such as the Endophthalmitis Vitrectomy Study (EVS), with values near to 30%.

## Conclusions

Intracameral bolus injection of cefazolin in cataract surgery demonstrated prophylactic efficacy in diminishing the rate of postoperative endophthalmitis in our hospital, at two doses of 1 mg in 0.1 ml solution.

Furthermore, a close relationship between with the Department of Microbiology, and the Infectious Diseases Committee of our Hospital is essential for developing a proper antibiotic prophylaxis.

Further studies are needed with a larger number of patients in order to fully determine the effectiveness of cefazolin and its non-toxicity at corneal endothelium level.

## Competing interests

The authors report no conflicts of interest. The authors alone are responsible for the content and writing of the paper. No financial support was received.

## Authors' contributions

PRA contributed to the study design, the researched data analysis, the Discussion, manuscript writing and supervision of the manuscript. IMM contributed to ophthalmologic data collection, contributed to the Discussion, made a critical review, and edited the manuscript. MAG contributed to the study design, systemic disease diagnosis and laboratory analysis interpretation, the researched data analysis and the Discussion. JRT contributed to the ophthalmologic data collection, and the researched data interpretation. JFB contributed to the statistical analysis, the researched data interpretation, and contributed to the interpretation of the study findings.

All authors read and approved the final manuscript.

## Appendix 1

### Hospitals that form the Barcelona Endophtalmitis Group (GEB). http://gebcn.org/geb.html

01. Hospital Sant Pau: Dr. Jesus Diaz

02. Hospital Clínic: Dra. Socorro Alforja - Dr. Joan Giralt

03. Hospital Bellvitge: Dr. Marc Rubio

04. Hospital Vall d'Hebron: Dr. Alex Fonollosa

05. Hospital General d'Hospitalet: Dra. Magela Garat

06. Hospital Germans Trias i Pujol (Can Ruti): Dr. Anglada

07. Hospital Parc Taulí: Dr. D. Muntaner - Dra. MªT. Sellarés - Dra. C. Guardia

08. Mutua de Terrassa: Dra. Silvia Freixes

09. IMAS (H. Esperança i Mar): Dr. Daniel Vilaplana - Dr. Poposki

10. Hospital St. Rafael: Dra. Ciprés

11. Hospital Viladecans: Dr. Sergi Sedó

12. Institut Català de la Retina: Dra. Sust

13. Institut Oftalmic de Barcelona: Dra. Laura Sararols

14. Instituto de Microcirugia Ocular: Dra. Isabel Nieto - Dra. Elena Arrondo

15. Hospital General de Catalunya: Dr. Rouras

16. Clínica Barraquer: Dr. Remberto Escoto

17. Hospital Espirit Sant: Dr. Rouras - Dr. Amias

18. Hospital Granollers: Dr. Jarek Hernecki

19. Hospital de Terrassa: Dr. Jose Juan Escobar - Dr. Jose Pradas

20. Institut Condal d'Oftalmologia (ICO): Dra. Sararols

21. Hospital Municipal de Badalona: Dra. Estela Barnola

22. OFTALMO: Dr. J. Cabot

23. Clínica de Vic i Hospital General de Vic: Dr. Manuel Amén

24. Consulta GMOftalmo: Dr. Gallofré

25. Hospital de Nens de Barcelona: Dr. J. Flors Despranell

26. Clinica Quiron: Dr. Aureli Rilo

27. Institut Oftalmológic Tres Torres: Dr. Emili Juarez

28. Hospital de Mollet: Dr. Francisco Goñi

29. Hosp. Universitari Josep Trueta: Dra. Flor Escalada

30. Hosp. Universitari Sant Joan de Reus: Dr. Pere Romero Dra. Isabel Mendez

31. Hosp. Universitari Joan XXIII Tarragona: Drs. Perez i Pardo

32. Hosp Verge de la Cinta de Tortosa: Dr. J Colome

33. Hosp Son Dureta, Palma de Mallorca: Dr. José Luis Olea

34. Clinica Sagrada Familia i Corachan: Dr. Carlos Martin

35. Clínica Plató: Dr. Joan Pujol - Dr. Sergi Miserachs

36. Institut Oftalmológic de Menorca: Dr. Pere Vilallonga

37. Hospital Sant Joan de Deu de Martorell: Dra. Mª Carmen del Aguila

38. CEM (Centro Especializado Microcirugía): Dra. Laura Sararols

## Pre-publication history

The pre-publication history for this paper can be accessed here:

http://www.biomedcentral.com/1471-2415/12/2/prepub
